# Exploring the effects of moxibustion on pathological remodeling in rats with myocardial ischemia-reperfusion injury based on mitochondrial cardiolipin protection

**DOI:** 10.3389/fphar.2026.1864586

**Published:** 2026-07-07

**Authors:** Chunmei Zhou, Senlei Xu, Qian He, Shilan Liang, Xuefeng Xia, Xue Chen, Yan Zuo, Shiyu Chen, Yihuang Gu, Hongru Zhang

**Affiliations:** 1 Key Laboratory of Acupuncture and Medicine Research, Ministry of Education, Nanjing University of Chinese Medicine, Nanjing, China; 2 Acupuncture and Tuina College, Nanjing University of Chinese Medicine, Nanjing, China; 3 Dan’an Shuyuan, Nanjing University of Chinese Medicine, Nanjing, China

**Keywords:** cardiac remodeling, cardiolipin, mitochondrial damage, moxibustion, oxidative stress

## Abstract

**Objective:**

Moxibustion has been shown to protect against myocardial ischemia-reperfusion injury (MIRI) by regulating mitochondrial function. Whether it preserves the structural integrity essential for this function remains unknown. Based on the critical role of cardiolipin (CL) in mitochondrial membranes, this study aimed to investigate whether CL serves as a novel target of moxibustion-mediated cardioprotection.

**Methods and results:**

A rat model of MIRI was established by ligating the left anterior descending (LAD) coronary artery for 30 min followed by 7 days of reperfusion. Moxibustion was applied to Neiguan acupoint (PC6) every other day during reperfusion. Moxibustion exerted significant cardioprotective effects by reducing interstitial fibrosis, alleviating cardiac hypertrophy, and improving cardiac function. Mechanistically, moxibustion attenuated oxidative stress-mediated mitochondrial injury, as shown by decreased ROS, 4-hydroxynonenal (4-HNE), total oxidative status (TOS), and oxidative stress index (OSI) levels, increased total antioxidant capacity (TAC), and preserved mitochondrial cristae structure. Moreover, moxibustion restored total CL levels, concomitant with downregulation of Acyl-CoA:lysocardiolipin acyltransferase-1 (ALCAT1) and reduction of cytosolic cytochrome c (Cyt c). Moxibustion also upregulated AMP-activated protein kinase (AMPK) phosphorylation, an effect reversed by Compound C which also largely abolished the cardioprotective effects, suggesting AMPK involvement.

**Conclusion:**

This study provides evidence for the involvement of the AMPK-oxidative stress-CL axis in moxibustion-mediated cardioprotection, highlighting the potential of moxibustion as a non-pharmacological intervention for ischemic heart disease.

## 1 Introduction

Currently, revascularization is widely regarded as the most effective therapeutic approach for acute ischemic heart disease, playing a pivotal role in improving survival rates (Rao et al., 2025). However, myocardial ischemia-reperfusion injury (MIRI) caused by it remains a challenge in clinical prognosis, which can lead to heart failure or other fatal outcomes (Heusch, 2020). To date, the clinical translation of pharmacological interventions targeting MIRI remains incomplete. Mitochondrial damage and oxidative stress during reperfusion may serve as fundamental drivers of subsequent molecular and cellular events associated with organ injury and pathological remodeling (Prag et al., 2023; Zhang et al., 2021). Notably, simple antioxidant therapy has not achieved ideal clinical benefits (Chen H. et al., 2025), highlighting the importance of myocardial protective strategies that target the fundamental pathological microenvironment of ROS generation and key mechanisms such as mitochondrial injury (Davidson et al., 2019; Lesnefsky et al., 2017).

Recent studies have demonstrated that moxibustion exerts therapeutic benefits in various diseases by improving mitochondrial respiration, autophagy, and dynamics (Chen X. et al., 2025; Yin et al., 2023). Previous studies have also shown that moxibustion on PC6 regulates mitochondrial function to attenuate MIRI (Zhang et al., 2019). Indeed, structural integrity is the prerequisite for functional recovery. Specifically localized to the inner membrane, cardiolipin (CL) is essential for maintaining mitochondrial structural integrity and functional homeostasis, and CL depletion is associated with increased cardiovascular risk, serving as an early characteristic biomarker of heart failure progression (Flori et al., 2025). A recent translational study demonstrated that VA-ECMO exacerbates myocardial injury in a closed-chest MIRI porcine model through acute CL depletion, whereas stabilizing CL significantly reduces infarct size (Swain et al., 2024). CL is highly susceptible to ROS attack, and its depletion destabilizes electron transport chain complexes, triggers cytochrome-c release, and activates apoptotic pathways (Bugger and Pfeil, 2020). AMP-activated protein kinase (AMPK) serves as the master redox-sensitive switch governing oxidative stress and mitochondrial homeostasis in MIRI (Cai et al., 2022). Studies have revealed that loss of AMPK activity in cardiomyocytes induces CL metabolic imbalance even under physiological conditions, and this imbalance can progressively develop into pathological remodeling and cardiac fibrosis (Tokarska-Schlattner et al., 2021; Grimbert et al., 2021). Notably, AMPK activation improves mitochondrial cristae structure by suppressing oxidative stress (Wang et al., 2021). Collectively, these findings suggest that moxibustion may protect against mitochondrial structural damage in MIRI by maintaining CL homeostasis through AMPK activation.

In the present study, we investigated the cardioprotective effects of moxibustion in a rat MIRI model. Our findings provide evidence that the AMPK-oxidative stress-CL axis contributes to these protective effects, highlighting mitochondrial CL as an underexplored target for moxibustion-based interventions in ischemic heart disease.

## 2 Materials and methods

### 2.1 Animals

Male Sprague-Dawley rats (240–260 g, SPF grade, Vital River Laboratory Animal Technology Co., Ltd., Beijing, China; license No. SCXK (Jing) 2021–0006) were housed under controlled conditions (temperature 25 °C ± 2 °C, humidity 50% ± 5%, 12:12 h light-dark cycle) with *ad libitum* access to food and water. Experimental procedures were approved by the Animal Ethics Committee of Nanjing University of Chinese Medicine (approval No. 202405A022) and conducted in accordance with the NIH Guide for the Care and Use of Laboratory Animals.

### 2.2 Experimental design and MIRI model

**FIGURE 1:**
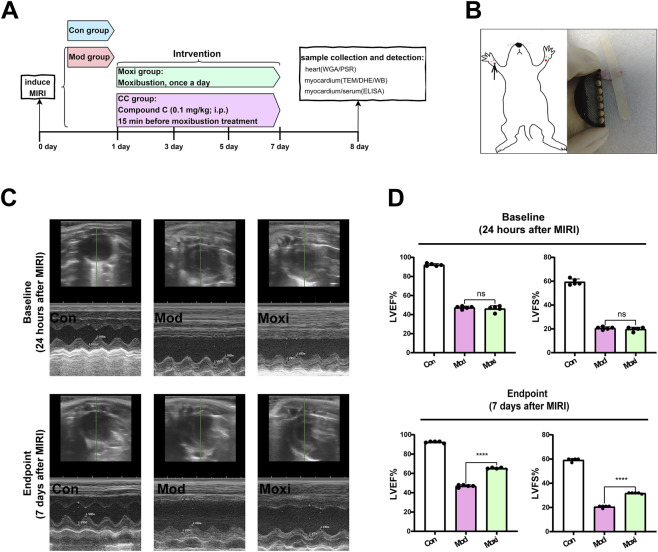
Moxibustion improved cardiac function in a rat model of MIRI. **(A)** The experimental protocol of this study. **(B)** The location and operation of moxibustion. **(C,D)** Representative M-mode echocardiographic images depicting left ventricular (LV) wall motion of rats in each group were detected at 24 h (baseline) and 7 days (endpoint) after MIRI, along with the corresponding LVEFs and LVFSs. ns, not significant; ****P < 0.0001.

Experimental design. After 7-day acclimatization, rats were randomized into four groups: Control (Con, n = 10), MIRI (Mod, n = 10), MIRI + Moxibustion (Moxi, n = 15), and MIRI + Compound C + Moxibustion (CC, n = 10). Group-specific interventions are detailed in Figure 1A and its legend.

Surgical procedure. Rats were anesthetized with 5% isoflurane (induction) and maintained with 2% isoflurane in 100% O_2_ (0.4 L/min) via endotracheal intubation on a heated surgical platform (37 °C ± 0.5 °C). Following left thoracotomy at the 4th intercostal space, the left anterior descending (LAD) coronary artery was ligated with a 6–0 Prolene suture for 30 min, followed by reperfusion. Successful induction of myocardial ischemia was confirmed by ST-segment elevation ≥50% on the electrocardiogram following LAD ligation, with ≥50% resolution upon reperfusion. Deaths occurred primarily during the ischemia-reperfusion procedure. The overall perioperative mortality rate was approximately 30%. All surviving animals met the inclusion criteria.

### 2.3 Moxibustion

Under 1.5% isoflurane anesthesia, rats were positioned supine with forelimbs extended and palms facing upward. The target site (PC6) was located on the anterior forelimb at the interosseous space between the radius and ulna, 3 mm proximal to the wrist joint (Figure 1B). Prior to each intervention, 5 mg of refined mugwort floss (derived from Artemisia argyi) was packed into a conical mold (as shown in Supplementary Material). Moxibustion was performed using 7 moxa cones placed on a dustproof net and ignited sequentially after each cone burned out per session. Treatments were administered on postoperative days 1, 3, 5, and 7.

### 2.4 Echocardiography

Transthoracic M-mode echocardiography was performed using a small-animal ultrasound system (Esaote, Italy) on days 1 and 7 post-surgery (Wu et al., 2022). Left ventricular dimensions were acquired in the parasternal long-axis view. Left ventricular end-diastolic diameter (LVEDd) and end-systolic diameter (LVESd) were measured, and ejection fraction (LVEF) and fractional shortening (LVFS) were calculated. Rats with an LVEF of 40%–50% on day 1 were enrolled in the study of moxibustion’s cardiac function following MIRI and served as the baseline level.

### 2.5 Tissue collection and processing

The rats were anesthetized with 5% isoflurane for induction, followed by maintenance with 3% isoflurane before sampling. Blood samples were collected from the abdominal aorta, and the rats were euthanized by cervical dislocation under deep isoflurane anesthesia. Death was confirmed by exsanguination and absence of corneal reflex and heartbeat. The collected blood samples were centrifuged at 3,000 × g for 15 min to obtain serum, which was then stored at −80 °C. Hearts were excised, weighed for heart weight (HW), and either fixed in 4% paraformaldehyde (PFA) or snap-frozen in liquid nitrogen. PFA-fixed tissues were dehydrated, embedded in paraffin, and sectioned at 5 μm using a microtome (Leica RM2125 RTS, Germany). Tibial length (TL) was measured, and HW/TL ratio was calculated (Zhang et al., 2024).

### 2.6 Transmission electron microscopy (TEM)

Fresh cardiac tissues from the infarct border zone were immediately fixed in 2.5% glutaraldehyde at 4 °C for 2 h, post-fixed in 1% osmium tetroxide for 2 h, dehydrated, and embedded in Epon 812 resin. Sections (60 nm) were cut with an ultramicrotome (Diatome Ultra 45°), stained with 2% uranyl acetate and 0.4% lead citrate, and examined under a transmission electron microscope (JEM-1400, JEOL, Japan).

### 2.7 Dihydroethidium (DHE) staining

Frozen sections (10 μm) were stained with dihydroethidium (Thermo Fisher Scientific) for ROS detection according to the manufacturer’s instructions.

### 2.8 Histological staining

Paraffin sections were stained with Sirius Red (SR) (Leagene, DC0041) for collagen deposition or Wheat Germ Agglutinin-XFD488 (WGA) (ATT Bioquest, 25,500) for cardiomyocyte membranes according to the manufacturers’ instructions. Images were acquired using a fluorescence microscope (Nikon, Japan) and analyzed with ImageJ.

### 2.9 Mitochondria and cytosol isolation

Mitochondria and cytosol were isolated using the Tissue Mitochondria Isolation Kit (Beyotime, C3606) according to the manufacturer’s instructions. Fresh heart tissue was minced, digested with trypsin, homogenized in isolation buffer with 1 mM PMSF, and subjected to differential centrifugation (600 × g for 5 min, followed by 11,000 × g for 10 min to pellet mitochondria). The post-mitochondrial supernatant was centrifuged at 12,000 × g for 10 min to obtain the cytosolic fraction. All steps were performed at 4 °C.

### 2.10 Biochemical analysis

*Immunoassays* were performed using commercial ELISA kits according to the manufacturers’ instructions. Serum N-terminal pro-B-type natriuretic peptide (NT-proBNP) and myocardial β-myosin heavy chain (β-MHC) were determined by the double-antibody method, while myocardial 4-hydroxynonenal (4-HNE) was measured by the competitive method (LAPUDA Biotechnology Co., Ltd.). CL in mitochondrial lysate (Section 2.9) was analyzed by the competitive ELISA method (ELK Biotechnology Co., Ltd.).

*Colorimetric assays* were performed to determine total antioxidant capacity (TAC) and total oxidant status (TOS) (Rynkiewicz-Szczepanska et al., 2026). TOS was assessed by the oxidation of Fe^2+^ to Fe^3+^ with H_2_O_2_ as the standard, and TAC by ABTS radical cation decolorization with Trolox as the standard. Results are expressed as μmol/L H_2_O_2_ or Trolox equivalents, respectively. The oxidative stress index (OSI) was calculated as the ratio of TOS to TAC.

### 2.11 Western blot (WB) assay

Western blotting was performed as described (Li S. et al., 2025). Mitochondrial and cytosolic fractions (Section 2.9) were analyzed for ALCAT1, cytochrome c, COX IV, and GAPDH. Whole-cell lysates were analyzed for total AMPK, phospho-AMPK (Thr172), and β-Actin. Primary antibodies: ALCAT1 (Bioss, bs-18190R, 1:1000), cytochrome c (Affinity, AF0146, 1:1000), COX IV (Affinity, AF5468, 1:1000), GAPDH (Proteintech, 60004-1-Ig, 1:20000), AMPKα (Proteintech, 10929-2-AP, 1:5000), phospho-AMPKα (Thr172) (Affinity, AF3423, 1:500), and β-Actin (Proteintech, 66009-1-Ig, 1:20,000). COX IV and GAPDH served as loading controls for mitochondrial and cytosolic fractions, respectively; β-Actin was used for whole-cell lysates. Signals were detected with ECL substrate (Tanon) and quantified using ImageJ.

### 2.12 Statistical analysis

Data are expressed as mean ± SD. Normality and homogeneity of variance were assessed by Shapiro-Wilk and Levene’s tests, respectively. Comparisons between two groups were performed using Student’s t-test as appropriate. Multiple group comparisons were analyzed by one-way ANOVA with Tukey’s *post hoc* test (equal variances) or Welch’s ANOVA with Games-Howell *post hoc* test (unequal variances). Statistical significance was set at P < 0.05. Analyses were performed using GraphPad Prism 8.0.

## 3 Results

### 3.1 Effect of moxibustion on cardiac function in rats with MIRI

Echocardiography was performed at baseline (24 h post-MIRI) and endpoint (7 days post-reperfusion) to assess left ventricular function. Baseline LVEF was comparable between the Mod and Moxi groups (ranging 40%–50%), confirming successful MIRI induction with uniform initial injury severity. At 7 days, the Mod group exhibited sustained cardiac dysfunction (LVEF 47.00% ± 1.23%, LVFS 20.60% ± 0.89%). In contrast, moxibustion treatment significantly improved cardiac function, with LVEF increasing to 65.20% ± 0.84% and LVFS to 31.80% ± 0.45%, indicating substantial recovery of contractile performance.

### 3.2 Effect of moxibustion on pathological cardiac remodeling in rats with MIRI

**FIGURE 2:**
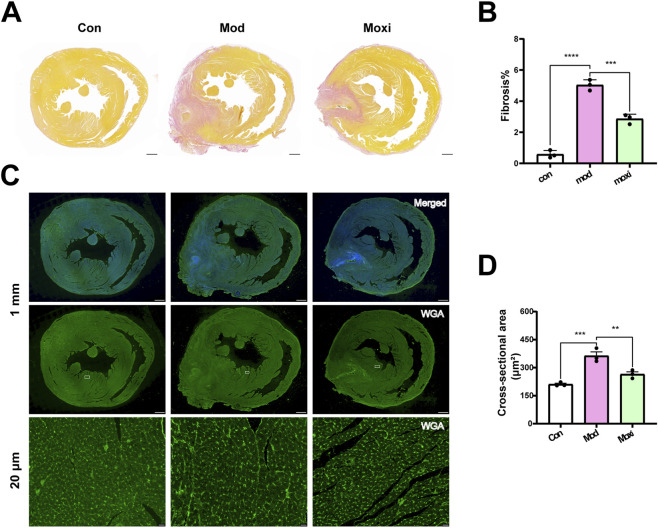
Moxibustion attenuated MIRI-induced cardiac hypertrophy and fibrosis in rats. **(A)** Sirius red (SR) staining showing interstitial collagen deposition (Scale bars = 1 mm). **(B)** Quantitative analysis of fibrosis percentage. **(C)** Representative immunofluorescence images showing merged (upper row) and WGA-stained (middle row) heart tissue at 1 mm magnification, and WGA-stained cardiomyocytes at 20 μm magnification (lower row). **(D)** Quantitative analysis of cardiomyocyte cross-sectional area. **P < 0.01; ***P < 0.001; ****P < 0.0001.

At 8 days post-reperfusion, hearts were harvested to evaluate structural remodeling. The Mod group displayed significant interstitial fibrosis (Figure 2A), cardiomyocyte hypertrophy (Figure 2B), compared with the Con group. Moxibustion treatment markedly reversed these pathological alterations, with reduced myocardial fibrosis and decreased cardiomyocyte cross-sectional area, compared with the Mod group. These findings demonstrate that moxibustion effectively mitigates adverse cardiac remodeling following MIRI.

### 3.3 Effects of moxibustion on oxidative stress and mitochondrial injury in rats with MIRI

**FIGURE 3:**
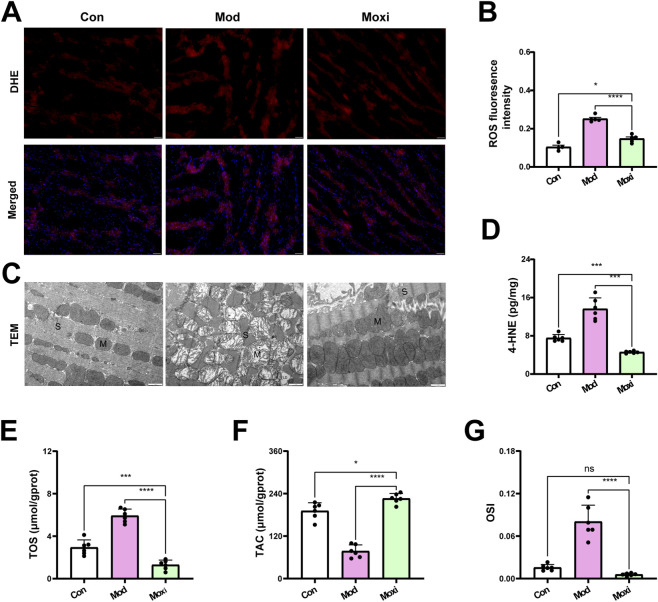
Moxibustion alleviated oxidative stress and mitochondrial injury in MIRI rats. **(A,B)** Representative images of dihydroethidium (DHE) staining (scale bars = 50 μm), along with corresponding quantitative analysis of ROS levels. **(C)** Representative transmission electron microscopy (TEM) images of left ventricular myocardium. Scale bar: 1 μm. M, mitochondria; S, sarcomeres; SR, sarcoplasmic reticulum; LD, lipid droplet. **(D)** The myocardial levels of 4-HNE. **(E–G)** Quantitative analysis of myocardial levels of total oxidant status (TOS), total antioxidant capacity (TAC), and oxidative stress index (OSI, that is TOS/TAC ratio). ns, not significant; *P < 0.05; ***P < 0.001; ****P < 0.0001.

A large amount of damaging ROS is formed during reperfusion, representing a critical mechanism of MIRI (Welt et al., 2024). We assessed myocardial redox status using DHE staining and biochemical assays (Figure 3). Moxibustion significantly reduced ROS generation (Figures 3A,B), decreased 4-HNE levels (Figure 3D), reduced TOS (Figure 3E), and lowered OSI (Figure 3G) compared with the Mod group. Notably, TAC was elevated above control levels (Figure 3F). Given that ROS overproduction is closely associated with mitochondrial structural damage (Hinton et al., 2024), we examined mitochondrial ultrastructure using transmission electron microscopy (TEM). TEM revealed severe mitochondrial swelling, cristae rupture, and vacuolization in the Mod group, whereas moxibustion preserved mitochondrial ultrastructure with intact cristae and reduced matrix swelling (Figure 3C).

### 3.4 Effect of moxibustion on cardiolipin homeostasis-related protein levels in the myocardium of MIRI rats

**FIGURE 4:**
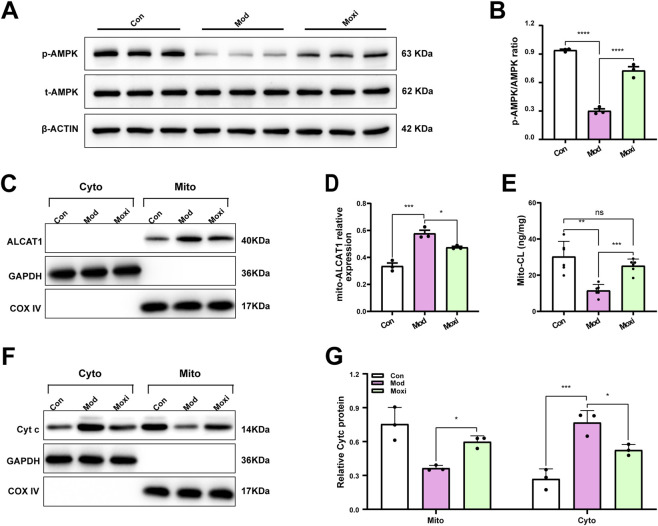
Moxibustion modulated mitochondrial cardiolipin and related molecular expression in MIRI rats. **(A,B)** Representative Western blot images and Quantitative summary data demonstrating the ratio of p-AMPK to AMPK in myocardium; **(C,D)** Representative Western blot images and quantitative analysis of ALCAT1 expression in mitochondrial and cytoplasmic fractions (COX IV and GAPDH as loading controls for mitochondrial and cytoplasmic proteins, respectively). **(E)** Cardiolipin (CL) content in myocardial mitochondria. **(F,G)** Representative Western blot images and quantitative analysis of cytochrome c (Cyt c) distribution in mitochondrial and cytoplasmic fractions (COX IV and GAPDH as loading controls). ns, not significant; *P < 0.05; **P < 0.01; ***P < 0.001; ****P < 0.0001.

Moxibustion alleviated oxidative stress and preserved mitochondrial cristae structure as described above. AMPK acts as a central regulator of mitochondrial function and is closely involved in the pathogenesis of cardiovascular diseases (Wu and Zou, 2020). Total AMPK protein expression remained unchanged across all groups, while phosphorylated AMPK (p-AMPK) levels were markedly increased in the Moxi group (Figures 4A,B). These findings suggest that AMPK activation contributes to the protective effects of moxibustion against mitochondrial oxidative damage. Given that mitochondrial CL is essential for membrane integrity yet highly susceptible to ROS-mediated oxidative damage (Paradies et al., 2018), we next investigated whether moxibustion regulates CL homeostasis. Compared with the Con group, the Mod group exhibited marked CL depletion, whereas moxibustion significantly restored total CL content. Because excessive ALCAT1 activation drives pathological CL remodeling and accelerates its degradation (Jia et al., 2021), we subsequently measured mitochondrial ALCAT1 expression to elucidate the underlying mechanism. Moxibustion significantly downregulated ALCAT1 expression (Figures 4C,D). Accordingly, cytosolic cytochrome c (Cyt c) release was remarkably reduced (Figures 4F,G), indicating restored mitochondrial membrane integrity. In summary, moxibustion increased AMPK phosphorylation while suppressing ALCAT1, contributing to the preservation of total cardiac CL levels following MIRI.

### 3.5 Inhibition of AMPK blocked the cardioprotection of moxibustion

**FIGURE 5:**
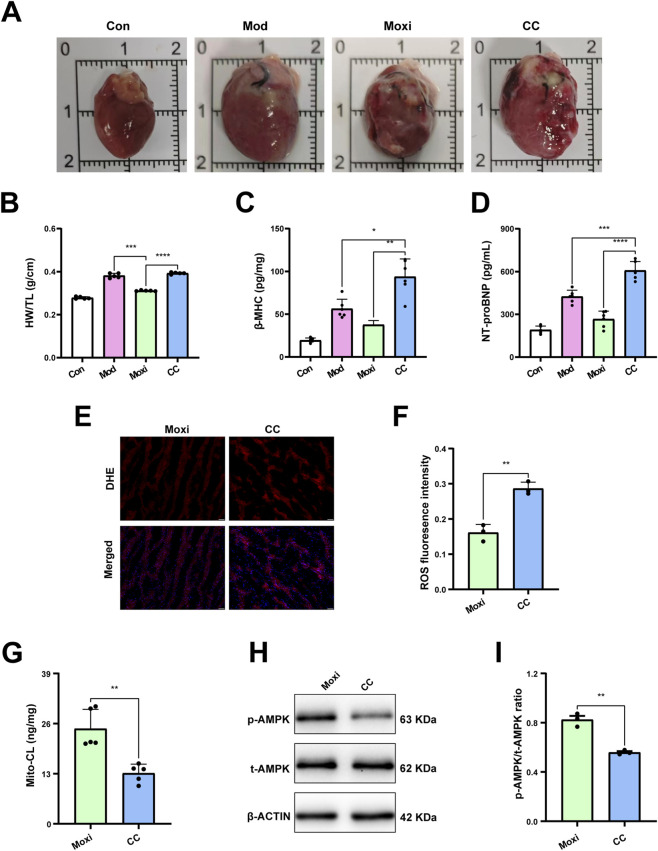
Pharmacological inhibition of AMPK abrogated the cardioprotective effects of moxibustion. **(A,B)** Representative gross morphology of hearts and heart weight-to-tibia length ratio (HW/TL). **(C,D)** The levels of myocardial β-MHC protein expression and serum NT-proBNP. **(E,F)** Representative images of DHE staining (scale bars = 50 μm), along with corresponding quantitative analysis of ROS levels. **(G)** Cardiolipin content in myocardial mitochondria. **(H,I)** Representative Western blot images and Quantitative summary data demonstrating the ratio of p-AMPK to AMPK in myocardium. Data in panels **(F,G,I)** were analyzed by Student’s t-test. *P < 0.05; **P < 0.01; ***P < 0.001; ****P < 0.0001.

To determine whether AMPK activation is necessary for the observed protective effects, the AMPK inhibitor Compound C (CC) was administered prior to moxibustion treatment. While moxibustion alone improved cardiac function and attenuated remodeling, CC co-treatment abolished these beneficial effects, as evidenced by reduced heart weight-to-tibia length ratio (HW/TL) (Figures 5A,B), and elevated levels of myocardial β-MHC and serum NT-proBNP (Figures 5C,D). Mechanistically, CC co-treatment significantly increased myocardial ROS accumulation (Figures 5E,F), aggravated mitochondrial CL depletion (Figure 5G) and reduced AMPK phosphorylation (Figures 5H,I) compared with moxibustion alone. These findings indicate that AMPK activation contributes to moxibustion-mediated suppression of oxidative stress, preservation of total CL levels, and protection against cardiac dysfunction following ischemia-reperfusion injury.

## 4 Discussion

Despite the established efficacy of revascularization for acute ischemic heart disease, the concomitant risk of MIRI continues to impair clinical outcomes (Welt et al., 2024; Martin, 2023). As no pharmacological agents have yet achieved reliable clinical application against MIRI, heat therapy has garnered attention as a viable alternative (Rodrigues et al., 2025). Notably, remote cardioprotective effects can be achieved through localized thermal stimulation without resorting to systemic whole-body heating (Wang et al., 2024). Unlike conventional heat therapy, moxibustion is a complex intervention that involves not only localized thermal stimulation but also combustion-derived volatile and bioactive components from moxa, as well as infrared radiation (Deng and Shen, 2013). As a traditional external therapy in Chinese medicine, moxibustion exerts comprehensive regulatory effects through these multifaceted stimuli(Xu et al., 2023) In accordance with meridian theory of traditional Chinese medicine, Neiguan (PC6) is closely associated with the cardiovascular system and has long been applied to treat chest pain, palpitations, and cardiac disorders related to myocardial ischemic injury (Tsou, et al., 2004; Zhang, et al., 2009). To our knowledge, this is the first study to link moxibustion on PC6 to the AMPK–oxidative stress–CL axis in the context of MIRI-induced cardiac dysfunction and pathological remodeling. While these findings are preliminary, they provide a rationale for further preclinical studies exploring the therapeutic potential of moxibustion in ischemic heart disease.

Mitochondrial dysfunction acts as a key initiating factor in the pathogenesis of MIRI (Bonora et al., 2022). It mediates oxidative stress, a core pathological process of MIRI triggered by a massive ROS burst during reperfusion (Li W. et al., 2025; Kalogeris et al., 2014), which disrupts the balance between ROS generation and endogenous antioxidant defense systems. This imbalance further exacerbates cardiomyocyte injury and aggravates mitochondrial dysfunction, forming a vicious cycle that ultimately promotes myocardial remodeling and cardiac dysfunction (Kura and Slezak, 2024; van der Pol et al., 2019). Accordingly, we further explored the regulatory effects and underlying mechanisms of moxibustion on oxidative stress and mitochondrial damage in MIRI. Our results demonstrated that moxibustion markedly alleviated oxidative injury induced by MIRI. Notably, regarding oxidative stress-related indicators, ROS levels in the Moxi group remained moderately higher than those in the Con group, while TAC was moderately elevated above control levels. This phenomenon embodies the TCM theoretical connotation of “struggle between healthy qi and pathogenic factors” and reflects the therapeutic principle of “strengthening healthy qi to eliminate pathogenic factors”. Collectively, these findings indicate that moxibustion remodels redox homeostasis toward an antioxidant-dominant state, enhances antioxidant reserve, and restrains the propagation of oxidative injury, thereby protecting cardiomyocytes against oxidative stress and preserving mitochondrial structural integrity.

CL homeostasis is essential for maintaining mitochondrial structural and functional integrity (Schlame and Ren, 2009). As a phospholipid crucial for bioenergetics, mitochondrial dynamics, autophagy, and apoptosis, CL is highly susceptible to oxidative damage owing to its high polyunsaturated fatty acid content and exclusive localization to the inner mitochondrial membrane (Lesnefsky and Hoppel, 2008; Xing et al., 2024). During MIRI, excessive ROS bursts trigger CL peroxidation (Xing et al., 2024), causing structural instability and increased vulnerability to phospholipase hydrolysis, and ultimately reducing functional CL levels (Paradies et al., 2018; Liu et al., 2017). Our study confirmed that moxibustion markedly improved mitochondrial ultrastructure, alleviating swelling, cristae disruption and vacuolization, and restored total CL content. ALCAT1 is a key enzyme in pathological CL remodeling. Prior evidence indicates that abnormal ALCAT1 activation mediates this process and mitochondrial dysfunction (Zhang et al., 2025). Its upregulation destabilizes CL configuration, accelerates oxidative modification and degradation, and eventually decreases mitochondrial CL abundance (Jia et al., 2021). Notably, oxidative stress can translationally upregulate ALCAT1 expression (Zhang and Shi, 2024), which explains the significant ALCAT1 elevation observed in our MIRI model. By suppressing oxidative stress, moxibustion downregulates ALCAT1 expression, thereby preserving total CL levels. As a signature phospholipid of the inner mitochondrial membrane, CL sustains mitochondrial function by anchoring Cyt c and stabilizing the electron transport chain (Ott et al., 2002). Concurrent with CL depletion, Cyt c release from mitochondria increases as early as 30 min after ischemia onset (Chen and Lesnefsky, 2006); this dual impairment disrupts electron transport chain function and exacerbates oxidative stress, ultimately promoting outer mitochondrial membrane permeabilization (Chai et al., 2025). Consistent with this pathological cascade, our findings demonstrate that moxibustion alleviates MIRI injury by inhibiting mitochondrial ALCAT1 overexpression, preserving total CL reserves, and suppressing cytoplasmic Cyt c release. These protective effects also embody the “treating the root cause of disease” principle of traditional Chinese medicine.

As aforementioned, AMPK inactivation in cardiomyocytes disrupts mitochondrial CL metabolism and contributes to pathological cardiac remodeling (Tokarska-Schlattner et al., 2021; Grimbert et al., 2021). AMPK is a central regulator of redox homeostasis that plays a crucial protective role in various cardiovascular diseases, including MIRI (Cai et al., 2022). Consistently, AMPK activation ameliorates mitochondrial injury by enhancing antioxidant capacity (Steinberg and Hardie, 2023). Combined with the regulatory effects of moxibustion on mitochondrial CL observed herein, We hypothesized that AMPK serves as a potential target whereby moxibustion regulates mitochondrial CL to protect against adverse remodeling following MIRI. In our study, moxibustion concurrently activated AMPK and inhibited ALCAT1 expression. Luo et al. (2026) reported that pioglitazone’s activation of AMPK phosphorylation and its subsequent reversal of oxidative stress, inflammation, and mitochondrial fission are blocked by Compound C. Hu et al. (2022) showed that icariin activates the AMPK/mTOR autophagy pathway to inhibit β-MHC, BNP, and ANP upregulation in ISO-induced cardiomyocyte hypertrophy, and this protective effect is abolished by Compound C (Hu et al., 2022). Elevated NT-proBNP reflects increased ventricular wall tension (González et al., 2022), while sustained mechanical stress activates fibroblasts and promotes collagen deposition, creating a pro-fibrotic pathomechanical environment (Silverman et al., 2025). During pathological remodeling, β-MHC is re-expressed at high levels due to its lower ATPase activity and reduced contractile efficiency, thereby accelerating hypertrophic decompensation (Han et al., 2025). To investigate the role of AMPK in the anti-remodeling effects of moxibustion, Compound C was applied in the present study. The results showed that Compound C significantly reversed the cardioprotective effects of moxibustion, including improved HW/TL, reduced β-MHC and NT-proBNP, suppressed ROS, preserved total CL, and restored AMPK phosphorylation. These findings suggest that AMPK acts as a critical mediator of moxibustion-induced mitochondrial cardiolipin protection and protection against adverse remodeling following MIRI.

Several limitations of the current study should be acknowledged. Firstly, while Compound C was used to pharmacologically inhibit AMPK, its known off-target effects preclude definitive conclusions regarding AMPK dependency; genetic approaches (e.g., siRNA, CRISPR/Cas9 knockdown/knockout) or complementary AMPK activators (e.g., AICAR, metformin) would be required to establish causality. Secondly, total cardiolipin levels were measured by ELISA without evaluation of oxidized cardiolipin species or cardiolipin remodeling; lipidomic analysis is warranted to fully characterize the mechanisms underlying cardiolipin preservation.

## Data Availability

The raw data supporting the conclusions of this article will be made available by the authors, without undue reservation.
