# Supplementary material for: Exploring the effects of moxibustion on pathological remodeling in rats with myocardial ischemia-reperfusion injury based on mitochondrial cardiolipin protection
Source: Front Pharmacol. 2026 Jul 7;17:1864586. doi: 10.3389/fphar.2026.1864586 (PMC13385720; doi:10.3389/fphar.2026.1864586)
Supplement: Supplementary file 1 [file Supplementaryfile1.docx]

Supplementary Material


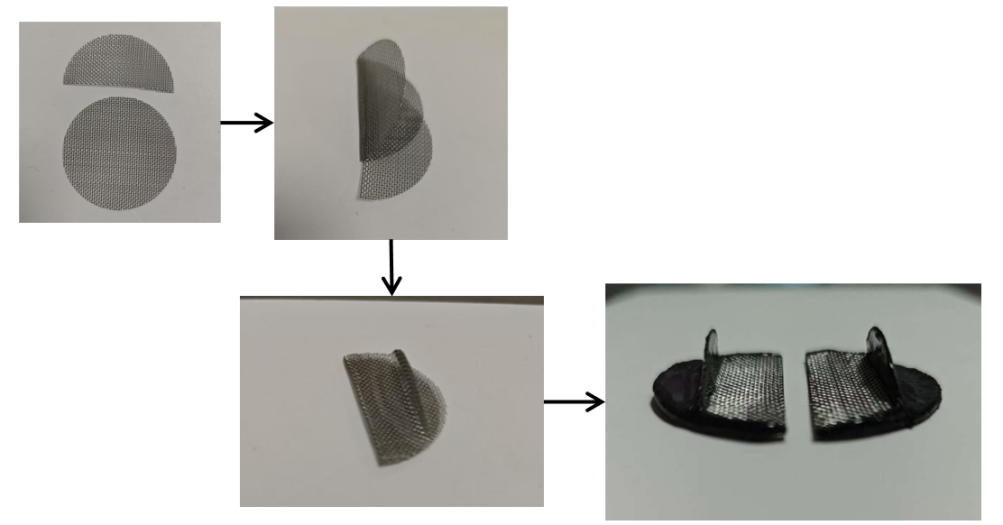


**Supplementary Figure 1.** dustproof net.


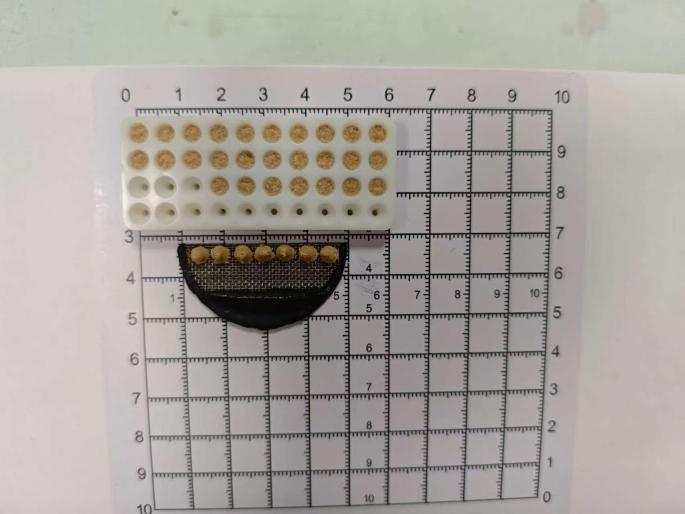


**Supplementary Figure 2.** moxa cone. A 5-mg portion of refined mugwort floss was shaped into a cone.


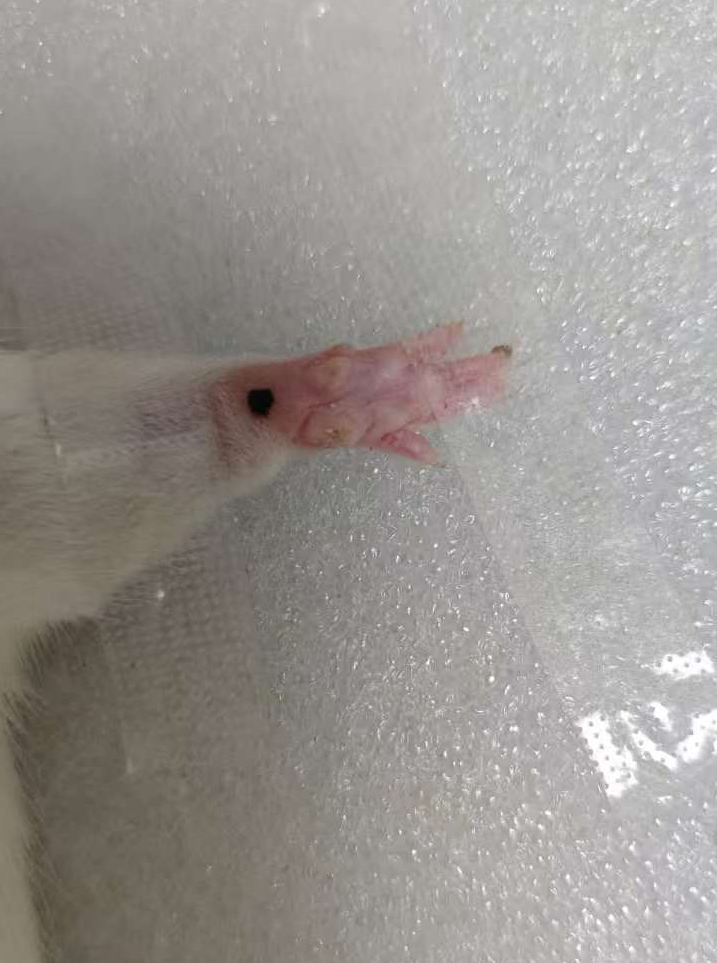


**Supplementary Figure 3.** moxibustion site. The thermal stimulation area was strictly confined to a fixed circular region, consistent with the size of the moxa cone.

**Supplementary Figure 4.** The skin-surface during moxibustion.


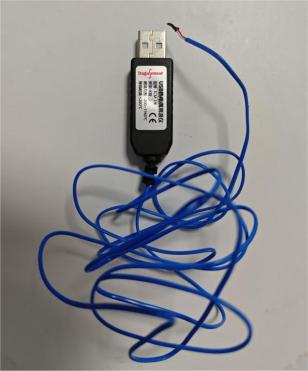


**Supplementary Figure 5.** Thermocouple.
